# Targeting phosphatases of regenerating liver (PRLs) in cancer

**DOI:** 10.1016/j.pharmthera.2018.05.014

**Published:** 2018-06-05

**Authors:** Min Wei, Konstantin V. Korotkov, Jessica S. Blackburn

**Affiliations:** Department of Molecular and Cellular Biochemistry, University of Kentucky, Lexington, KY, United States

**Keywords:** Protein tyrosine phosphatase, Metastasis, Theinopyridone, Structure

## Abstract

The phosphatase of regenerating liver (PRL) family, also known as protein tyrosine phosphatase 4A (PTP4A), are dual-specificity phosphatases with largely unknown cellular functions. However, accumulating evidence indicates that PRLs are oncogenic across a broad variety of human cancers. PRLs are highly expressed in advanced tumors and metastases compared to early stage cancers or matched healthy tissue, and high expression of PRLs often correlates with poor patient prognosis. Consequentially, PRLs have been considered potential therapeutic targets in cancer. Persistent efforts have been made to define their role and mechanism in cancer progression and to create specific PRL inhibitors for basic research and drug development. However, targeting PRLs with small molecules remains challenging due to the highly conserved active site of protein tyrosine phosphatases and a high degree of sequence similarity between the PRL protein families. Here, we review the current PRL inhibitors, including the strategies used for their identification, their biological efficacy, potency, and selectivity, with a special focus on how PRL structure can inform future efforts to develop specific PRL inhibitors.

## 1. Introduction

Protein tyrosine phosphatases (PTPs) are a large family of enzymes that catalyze the removal of phosphate groups that are attached to tyrosine residues on their substrates. PTPs, together with protein tyrosine kinases (PTKs), precisely maintain the appropriate phosphorylation level of proteins, which is critical for normal cellular functions.

The aberrant phosphorylation of proteins is implicated in many human diseases, including cancer, inflammatory diseases, and diabetes/obesity (Z. Y.A Zhang, 2017), suggesting both PTPs and PTKs are potential therapeutic targets. PTK inhibitors have achieved clinical success and become the standard of care in several types of cancer, including Afatinib for non-small-cell lung cancer (Y.-L. Wu et al., 2014) and Imatinib for chronic myeloid leukemia (Comert, Baran, & Saydam, 2013). Conversely, PTPs have not received attention as therapeutic targets until the past decade, due to misconceptions that phosphatases are only tumor suppressors or that they lack regulatory roles in disease (Lazo & Sharlow, 2016. However, accumulating evidence has shown that phosphatases are suitable therapeutic targets in cancer. For example, protein tyrosine phosphatase 1B (PTP1B) is reported to play a tumor-promoting role in prostate and colorectal cancer (Lessard et al., 2012), and high PTPB1 expression is associated with poor prognosis in colorectal cancer patients (Hoekstra et al., 2016; Lessard et al., 2012). Additionally, protein tyrosine phosphatase SHP2 increases tumor progression and maintains tumor-initiating cells in breast cancer (Aceto et al., 2012; Hu, Li, Gao, Wei, & Yang, 2017). Consequently, the interest in exploring phosphatases as drug targets to treat cancer has risen sharply in the last decade.

## 2. PRLs as oncogenic phosphatases

The protein tyrosine phosphatase 4A (PTP4A) family, commonly known as phosphatase of regenerating liver (PRLs) are dual-specificity phosphatases, which can act on both tyrosine residues and serine/threonine residues (Bessette, Qiu, & Pallen, 2008). PRLs are largely considered oncogenic phosphatases that play critical roles in tumor progression and metastasis across a variety of human cancers. PRL-3 is the most well-studied of the PRLs, and is highly-expressed in many types of solid tumors and leukemia, reviewed in detail elsewhere (Bollu, Mazumdar, Savage, & Brown, 2017; Campbell & Zhang, 2014; Stephens, Han, Gokhale, & Von Hoff, 2005). Importantly, metastatic lesions in many of these solid cancers expressed PRL-3 at much higher levels than the primary tumor, and high PRL-3 expression was often correlated with poor patient prognosis (Beekman et al., 2011; Dai, Lu, Shou, & Li, 2009; Mayinuer et al., 2013; Qu et al., 2014; Radke et al., 2006; Ren et al., 2009; Saha et al., 2001), suggesting a causative role for PRL-3 in cancer progression. A direct contributing role for PRL-3 in cancer has been demonstrated by over-expression and knock-down of PRL-3 in normal or cancer cell lines. For example, human cell lines transfected with PRL-3, including human melanoma, breast, lung and colorectal cancer, exhibited increased oncogenic properties compared to control, including increased motility, migration, invasion and proliferation *in vitro*. PRL-3 expression significantly enhanced tumor progression and metastasis after transplantation of the transfected cells in mice (Guo et al., 2004; Hardy, Wong, Muller, Park, & Tremblay, 2010; X. Wu et al., 2004). Conversely, PRL-3 knock-down led to decreased cell proliferation, migration, and invasion of melanoma, gastric, ovarian, lung cancer cell lines *in vitro* and inhibited primary tumor proliferation and metastasis in mouse cancers or xenograft models (Achiwa & Lazo, 2007; Hardy et al., 2010; Kato et al., 2004; Li et al., 2006; Polato et al., 2005; Qian et al., 2007; Y. Wang & Lazo, 2012).

Similarly, both PRL-1 and PRL-2 are reported to have oncogenic roles in cancer, but these are not well-defined. High PRL-1 expression was observed in cervical (Dong, Sui, Wang, Chen, & Sun, 2014) and gastric cancers (Dumaual et al., 2012) and intrahepatic cholangiocarcinoma (Liu et al., 2016). PRL-1 expression was correlated with poor patient prognosis in hepatocellular carcinoma (Jin et al., 2014) and prostate cancer (Shinmei et al., 2014). PRL-2 expression was significantly increased in breast cancer (Hardy et al., 2015) and hepatocellular carcinomas (Dumaual et al., 2012). Inconsistently, in situ hybridization and immunohistochemistry showed that PRL-1 expression was lower in ovarian, breast, and lung cancers and PRL-2 was significantly down-regulated in kidney carcinomas compared to normal tissue (Dumaual et al., 2012). However, the number of cases examined in this study was limited, and further research needed to validate the expression level of PRL-1 and PRL-2 in these cancer types.

Studies of PRL-1 or PRL-2 over-expression or knock-down in cell lines show that these PRLs may have similar functions as PRL-3. For example, PRL-1 overexpression in chinese hamster ovary (CHO) cells led to increased cell motility and invasiveness *in vitro*. The injection of those cells in nude mice induced lung tumor and liver metastasis, similar to the effects of PRL-3 overexpression in CHO cells (Zeng et al., 2003). The D27 hamster pancreatic ductal epithelial cells that ectopically overexpress PRL-1 or PRL-2 showed loss of contact inhibition *in vitro* and induced tumor growth in nude mice (Cates et al., 1996). Different mouse mammary tumor–derived cell lines that overexpress PRL-2 showed increased anchorage-independent growth and cell migration. In addition, injection of DB-7 mammary cancer cells with PRL-2 overexpression into the mouse mammary fat pad increased tumor growth (Serge Hardy et al., 2010). Finally, PRL-2 knock-down reduced the anchorage-independent growth and cell migration of human metastatic MDA-MB-231 breast cancer cells and reduced the cell migration and invasion of human A549 lung cancer cells, which can be rescued by co-transfecting an siRNA resistant PRL-2 (Y. Wang & Lazo, 2012).

While the experimental evidence above clearly establishes the oncogenic role for the PRL phosphatase family in cancer cells, PRLs may also play an important role in the tumor angiogenesis. For example, PRL-3 mRNA was detected in endothelial cells within a colon cancer metastasis (Bardelli et al., 2003) and was increased 6-fold in breast tumor endothelium compared to surrounding epithelial cells (Parker et al., 2004). Overexpression of PRL-3 in human microvascular endothelial cells (HMVEC) *in vitro* enhanced endothelial tube formation (Rouleau et al., 2006) and endothelial cell migration (Parker et al., 2004). Additionally, PRL-3 knock-out in mice led to decreased microvessel density in colon tumor tissues compared with wild type controls. In addition, vascular cells isolated from PRL-3-null mice were less invasive and migratory *in vitro*, compared with wild type cells (Zimmerman et al., 2014). Further studies are needed to definitively link PRL-3 to angiogenesis in the cancer setting, and the role of PRL-3 in other migratory cells within the tumor microenvironment, such as fibroblasts and immune cells, remains to be defined.

## 3. PRL substrates

Despite the relatively well-established functional role of PRLs in cancer progression, the molecular mechanisms through which PRLs promote cancer cell proliferation, invasion and metastasis are largely undefined. Mechanistically, PRLs have been shown to be involved in several major signaling pathways, including regulation of p53, PTEN/PI3K/Akt, Src/ERK1/2, Rho family GTPases and adhesion proteins including integrin, E-Cadherin and matrix metalloproteases (Campbell & Zhang, 2014; Rios, Li, & Kohn, 2013).

Identification of the substrates of phosphatases is highly challenging due to the complicated substrate profiles that may include proteins, lipids, and carbohydrates, as well as the transient interaction between most phosphatases and their substrates (Fahs, Lujan, & Kohn, 2016). This difficulty in identifying substrates is best reflected by the fact that there are only 305 protein substrates and 89 non-protein substrates identified for 194 human phosphatases according to the DEPOD database (http://depod.bioss.uni-freiburg.de/br_s.php) as of April 2018. In contrast, there are 5092 protein substrates for 518 protein kinases according to the RegPhos (http://140.138.144.141/~RegPhos/index. php). It may be even more challenging to identify PRL substrates, as the catalytic pocket of PRLs are more shallow and wider compared to other PTPs (Kozlov et al., 2004), making substrate trapping difficult. Consequently, only a few direct substrates have been suggested for PRLs, including phosphatydilinositol (4,5) bisphosphate [PI(4,5)P2] (McParland et al., 2011), Ezrin (Forte et al., 2008), Stathmin (Zheng et al., 2010), Keratin 8 (Mizuuchi, Semba, Kodama, & Yokozaki, 2009), Integrin α1 (Peng et al., 2006), Elongation factor 2 (Orsatti et al., 2009) and Nucleolin (Semba, Mizuuchi, & Yokozaki, 2010). Different strategies have been used to identify these substrates, including proteomics (Zheng et al., 2010), a yeast two-hybrid system (Peng et al., 2006), immunoprecipitation using wild-type and catalytically inactive PRL (Semba et al., 2010), *in vitro* dephosphorylation assays (McParland et al., 2011) and comparative studies of the phosphorylation of proteins in the cells that overexpress wild-type PRL or catalytic inactive PRL (Forte et al., 2008). However, most of these suggested substrates have not been validated in the signaling pathways that are affected by PRLs.

A new, non-phosphatase role for PRLs in cancer was recently proposed, whereby PRL binds to magnesium transporters of the cyclin M (CNNM) family to increase intracellular magnesium concentration by either increasing its influx or blocking its efflux. High intracellular magnesium concentration has been shown to contribute to tumorigenesis and progression based on studies on cultured cells, animal models, and human samples (Castiglioni & Maier, 2011). A xenograft tumor assay demonstrated that breast cancer cells that overexpress CNNM3 are more oncogenic compared with CNNM3 G433D, a mutant without ability to bind to PRL-2 (Hardy et al., 2015; Kostantin et al., 2016). Similar results were achieved by using another binding-deficient CNNM3 mutant to inhibit PRL-2-CNNM complex formation (Kostantin et al., 2016). PRL-3-CNNM4 interaction was reported to block magnesium efflux and promote colon cancer development in a mouse model (Funato et al., 2014). CNNMs are not phosphorylated substrates of PRLs (discussed in detail in Section 6.3), suggesting that the role for PRLs in cancer may extend beyond their phosphatase activity.

Despite the ongoing uncertainty regarding PRL substrates, these studies also suggest that PRLs are important therapeutic targets across many different cancer types, whether they function as a phosphatase and/or pseudophosphatase. Extensive efforts have been put in resolving their structures and inhibitor development. PRL-1 and PRL-3 protein structures have been resolved by using crystallography and nuclear magnetic resonance (NMR), respectively. Several different groups of PRL inhibitors have recently been identified. However, the selectivity and/or potency of the inhibitors is still limited, due to their predicted ability to act upon other phosphatases, leading to unwanted side effects. In addition, inhibitors that could target individual PRLs would be incredibly useful in dissecting the biological functions of each PRL. Here, we discuss the progress toward defining the structure of PRL-1 and PRL-3 (the PRL-2 apo structure is not resolved yet) and how those data can be utilized for the development of better PRL inhibitors. We will also summarize the currently available PRL inhibitors and compare their selectivity, potency and validated biological functions.

## 4. Homology among PRLs

PRL-1 was the first identified PRL, discovered in 1991 as one of the immediate-early genes up-regulated in regenerating rat liver after partial hepatectomy (Mohn et al., 1991). Later, the sequence analysis of PRL-1 identified a PTP signature motif, valine-histidine-cysteine-(any amino acid)-arginine (VHC(X)_5_R), but with no homology to other PTPs outside this signature sequence. *in vitro* phosphatase assays using a generic DiFMUP (6,8-Difluoro-4-Methylumbelliferyl Phosphate) substrate demonstrated that PRL-1 had phosphatase activity. Therefore, PRL-1 emerged as the first in a new class of PTPs (Diamond, Cressman, Laz, Abrams, & Taub, 1994).

**Fig. 1.**
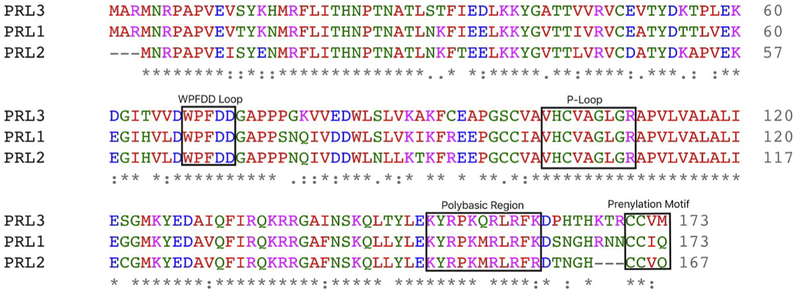
PRL phosphatases are highly homologous. The P-Loop and WPFDD Loop are critical to phosphatase activity. The prenylation motif targets PRLs to the plasma membrane. The polybasic region facilitates PRLs binding to the plasma membrane.

Later, PRL-2 and PRL-3 were identified based on a sequence homology search in the murine expressed sequence tags database (Zeng, Hong, & Tan, 1998). In humans, PRL-1 and PRL-2 are most similar in amino sequence, sharing 87% homology, while PRL-1 and PRL-3 exhibit 79% homology, and PRL-2 and PRL-3 are 76% homologous (Fig. 1) (Rios et al., 2013; Stephens et al., 2005).

The genes that encode PRL-1, PRL-2 and PRL-3 are found on chromosome 6q12, 1p35 and 8q24.3, respectively. PRL-1 and PRL-2 mRNA expression was ubiquitously detected across almost all normal human tissues and major organ systems (Dumaual, Sandusky, Crowell, & Randall, 2006), while PRL-3 expression was detected primarily in the heart, skeletal muscle, vasculature and brain (Zeng et al., 1998). The normal cellular functions of PRLs have not yet been identified.

PRL proteins have a small molecular weight, at 22 kDa, with 173 amino acids in PRL-1 and PRL-3, and 167 amino acids in PRL-2 (Stephens et al., 2005). Sequence alignment of PRL-1, PRL-2 and PRL-3 shows that they all carry the conserved catalytic PTP motif VHC(X)_5_R, also known as a P-loop, a trypotophan-proline-phenylalanine-aspartate-aspartate (WPFDD) loop, polybasic region and cysteine-aliphatic amino acid- (any amino acid) (CAAX) prenylation motif (Fig. 1), the functions of which are highlighted below.

**Fig. 2.**
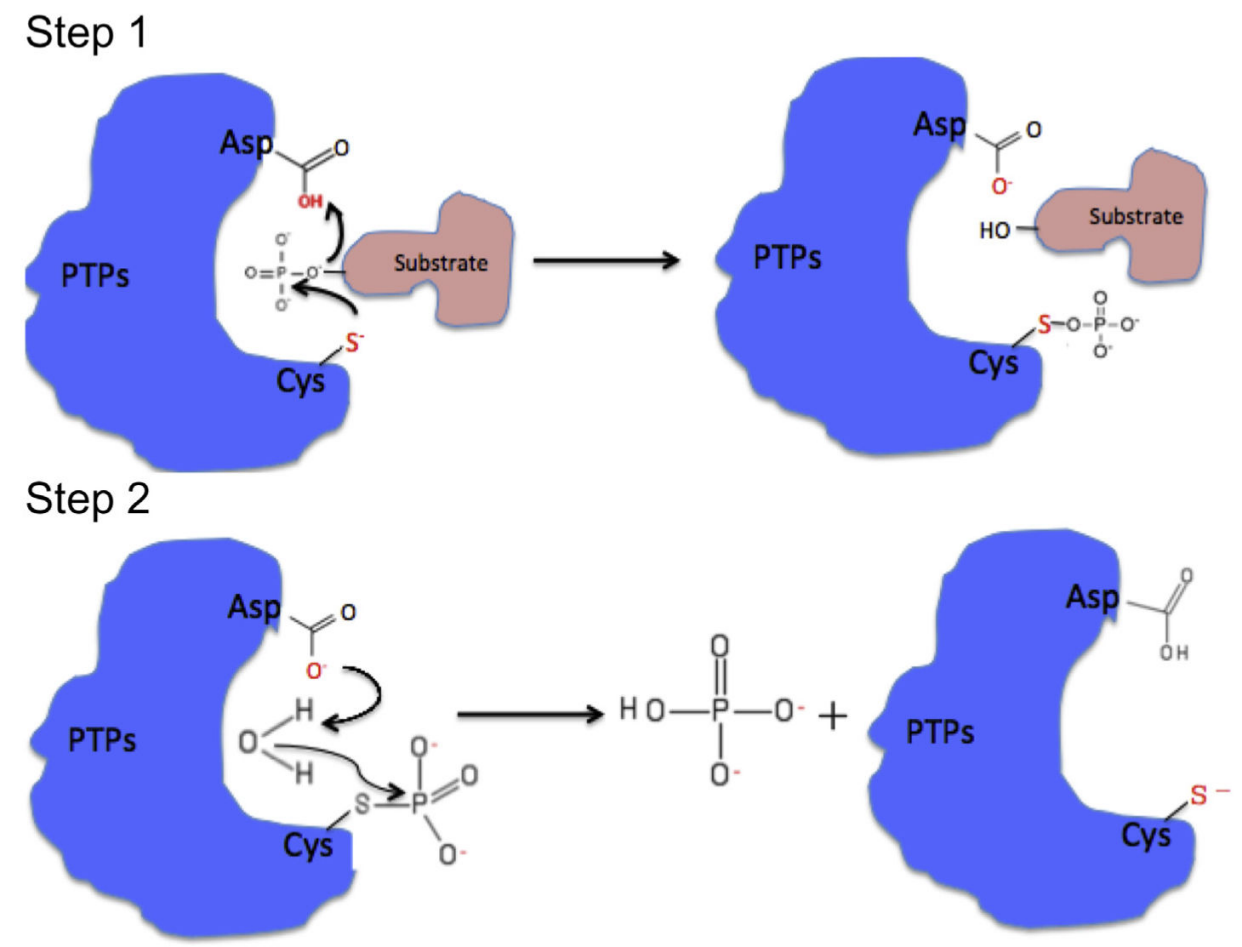
Two-step catalytic mechanism of PTPs. PRLs share a canonical two-step catalytic mechanism with other PTPs. In step one, the thiolate anion of the Cysteine residue in the P-loop acts as a nucleophile, attacking the phosphate group on the substrate then forming a thiophosphoryl enzyme intermediate, and the second aspartate reside in the WPFDD loop acts as a general acid by donating a proton to the leaving group in the substrate. In step two, the same aspartate residue acts as a general base by activating a water molecule that can hydrolyze the enzyme-phosphate complex and then release the phosphate group.

Similar to other PTPs, the cysteine residue in the P-loop (Cys^104^ in PRL-1 and PRL-3 or Cys^101^ in PRL-2) acts as a nucleophile during phosphorylation, forming a thiophosphoryl enzyme intermediate (Fig. 2). C104S mutation was shown to abolish PRL enzymatic activity *in vitro* (Kozlov et al., 2004; H. Zhang et al., 2017) or its metastatic activity in a xenograft mouse model (Guo et al., 2004). The arginine residue in the P-loop (Arg^110^ in PRL-1 and PRL-3 or Arg^107^ in PRL-2) facilitates substrate binding by interacting with the phospho-tyrosine of substrates. An R110A mutation in PRL-3 completely abolished its phosphatase activity *in vitro* (H. Zhang et al., 2017). The second aspartate residue in the WPFDD loop (Asp^72^ in PRL-1 and PRL-3 or Asp^69^ in PRL-2) acts as a general acid by donating a proton to the substrate at the first step and a general base by activating a water molecule in the second step, promoting formation and hydrolysis of the enzyme intermediate (Fig. 2). D72A mutation has been reported to decrease PRL catalytic activity (Kozlov et al., 2004; J. Wang, Kirby, & Herbst, 2002). Therefore, both the P-loop and WPFDD loop are critical for PRL phosphatase activity.

The CAAX motif, also known as prenylation motif, at C-terminus of PRLs is unique to PRLs and not found in other PTPs. This feature is important for PRL subcellular localization (Zeng et al., 2000). The polybasic region preceding CAAX motif (between 151 and 161 residues in PRL-1 and PRL-3 or between 148 and 158 in PRL-2) facilitates PRLs binding to the membrane by interacting with negatively charged phospholipids in the membrane (Bessette et al., 2008; Sun et al., 2005). Overexpressed N-terminal Myc-tagged PRL-1, PRL-2, and PRL-3 in CHO cells were all shown to localize on the plasma membrane and early endosome using immunofluorescent microscopy and electron microscope immunogold labeling. Inhibition of prenylation of PRL-1, PRL-2, and PRL-3 using selective farnesyltransferase inhibitor FTT-277 led to redistribution of all the PRLs into the nucleus. Similarly, the truncated PRL-2 without the CAAX motif was localized in the nucleus. The redistribution of PRLs between plasma membrane and nucleus may play a role of functional regulation of PRLs (Zeng et al., 2000).

The alanine residue following the arginine residue in the P-loop also differentiates PRLs from other PTPs (Fig. 2). Serine or threonine, which is believed to play an important role in the hydrolysis of the phosphoenzyme intermediate, occupies this position in most PTPs. Consistently, a mutation of PRL-1 that replaces this alanine with a serine (A111S) showed increased activity toward synthetic substrate (Sun et al., 2005). The presence of alanine instead of serine/threonine in PRLs may therefore contribute partially to the low phosphatase activity of PRLs *in vitro*. The residues in P-loop of PRL-3, Val^105^-Ala-Gly-Leu-Gly^109^, is highly hydrophobic compared with other phosphatases (Kozlov et al., 2004), which suggests different substrate selectivity. This feature suggests the possibility of developing hydrophobic high-affinity competitive PRL inhibitors with better cell permeability, as other PTP competitive inhibitors are more likely to be highly charged due to a charged active site.

## 5. Current PRL inhibitors and their use as anti-cancer agents

As the contributing role of PRLs in tumor progression and metastases is now widely accepted, there has been great interest in developing specific PRL inhibitors as novel anti-cancer reagents. However, the conservative active site of PRLs with other PTPs and the high percentage of identical primary sequence among PRLs present obstacles for developing small molecules that target the PRL family (Rios et al., 2013). Consequently, the currently available PRL inhibitors have low selectivity, exhibiting inhibitory effects against other PTPs or all three PRLs (Table 1). As several PTPs, such as PTEN, are well known as tumor suppressors, it is critical to specifically target the oncogenic PRLs while sparing the tumor-suppressing PTPs.

Table 1 describes the currently available PRL inhibitors, which were identified or developed by high-throughput screening, virtual screening and/or SAR studies, and natural product screening.

A high-throughput screen of the Roche chemical library for molecules that inhibit PRL phosphatase activity against a peptide substrate identified thienopyridone, which showed selectivity for PRLs over 11 other phosphatases, including tyrosine phosphatases and dual-specificity phosphatases such as PTP1B, SHP2, and CD-45 (Daouti et al., 2008). Thienopyridone was shown to significantly inhibit tumor cell anchorage-independent growth in soft agar and migration, and induce anoikis via induction of the p130Cas cleavage in a p53-independent manner (Daouti et al., 2008). A structural-activity-relationship (SAR) study of thienopyridone aiming to increase its stability and reduce its potential toxicity led to the development of iminothienopyridinedione 13 (JMS-053), which is a photooxygenation product of thienopyridone and showed a 10-fold increase in potency compared to thienopyridone (McQueeney et al., 2018; Salamoun et al., 2016). In addition, JMS-053 exhibits anti-tumor activity on drug-resistant ovarian cancer in a murine xenograft model at concentration as low as 0.1 μM. While JMS-053 is the most potent and specific PRL inhibitor yet developed, neither thienopyridone nor JMS-053 are specific among three PRLS. Although these compounds may still be beneficial, since all PRLs appear to have oncogenic effects and it may not be necessary to selectively target them, until the normal functions of PRLs are known, it may be difficult to bring these compounds to the clinic.

Another group of PRL inhibitors that were developed based on high-throughput screening are rhodanine and its derivatives. The rhodanine skeleton was identified during a high-throughput screening of the Korea Chemical Bank for PRL-3 inhibitors. Rhodanine derivatives were subsequently synthesized and cellular based assays were used to screen for specific and potent PRL inhibitors. The most potent derivatives, compound 5e, reduces invasiveness of B16F10 melanoma cells *in vitro*. Further SAR study identified two other potent rhodanine derivatives, CG-707 and BR-1, could recover the phosphorylation level of potential PRL-3 substrates, such as Ezrin and Cytokeratin 8 (Min et al., 2013). CG-707 and BR-1 significantly suppressed the migration and invasion of cancer cells with high PRL-3 expression, but had minimal effects on the cells that expressed low levels of PRL-3, suggesting that the antitumor effect of these compounds was correlated with their ability to block PRL-3 activity (Min et al., 2013). Mechanistic studies revealed that CG-707 could regulate the expression of EMT markers, increasing E-cadherin expression while decreasing Snail expression. Currently BR-1 (CAS No.893449–38-2) is commercially available. However, extensive studies have reported that the rhodanine scaffold is generally non-selective and can react with diverse proteins, so it is not an ideal lead compound for drug development (Mendgen, Steuer, & Klein, 2012; Tang, Lee, Packiaraj, Ho, & Chai, 2015; Tomasic & Masic, 2009).

The resolved PRL-1 crystal structures and PRL-3 NMR structures make virtual screening of PRL inhibitors possible. A novel PRL-3 inhibitor, 2-cyano-2-ene-ester, was identified by ligand-based screening for thienopyridone – shape–like molecules among 641,485,760 conformers generated from 3,472,461 lead-like molecules in the Zinc database (Hoeger, Diether, Ballester, & Kohn, 2014). The subsequent SAR based on this compound led to the identification of Analog 3, which shows acceptable selectivity for PRL-3 compared to the phosphatases PTP1B, TCPTP and VHR, but has no selectivity for PRL-3 over PRL-1 and PRL-2. Biologically, Analog 3 inhibits migration of cells that express PRLs in a dose-dependent manner and does not affect proliferation of HEK cells at 50 μM, implicating low cytotoxicity.

Zhong-Yin Zhang's laboratory developed a new strategy to develop PRL inhibitors, disrupting PRL trimerization. PRLs are the only PTPs currently known to form trimers, therefore small molecules that can disrupt PRL trimerization would be specific PRL inhibitors. They performed sequential structure-based virtual screening to sample 560,000 compounds of Asinex and ChemBridge subsets in the ZINC database, searching for the molecules that can bind to PRL-1 trimer interface. Fifty-six compounds returned by virtual screening were evaluated for their capability to disrupt PRL-1 trimer formation by *in vitro* or *in vivo* cell-based cross-linking assays. Compound-43 and several analogs were identified as trimer disruptors. Compound-43 specifically inhibits cell proliferation and migration of PRL-1 overexpressing cells and suppresses MeWo human melanoma cells proliferation and migration. Importantly, it also inhibits melanoma xenograft tumor growth (Bai etal., 2016).

The FDA-approved drug pentamidine has been used in clinic as an anti-protozoa drug to treat leishmaniasia, a tropical disease, for several years. The fact that pentamidine has similar anti-leishmania action to sodium stibogluconate, which is known to have anti-cancer activity via PTP inhibition, prompted the investigation of pentamidine's effect on PTP activity. *in vitro* enzymatic assays showed that pentamidine inhibited the phosphatase activity of both recombinant and ectopically expressed PRLs. More importantly, pentamidine inhibited *in vitro* growth of multiple cancer cell lines expressing endogenous PRLs, including melanoma, prostate, ovarian, colon and lung carcinomas. It also inhibited growth of human melanoma WM9 cells in nude mice. While pentamidine shows potent effects on PRLs, it also inhibited PTP1B phosphatase activity. Its selectivity for PRLs over other PTPs has not yet been tested (Pathak et al., 2002).

Natural products have also been shown to have PRL inhibitory activity. These include ginkgetin and sciadopitysin, which were identified in the extract of the branches of *Taxus cuspidata*, anthraquinone compounds extracted from the roots of *Rubia akane*, and curcumin from the spice turmeric. However, the specificity of these compounds has not been tested on other PTPs (Choi et al., 2006; Moon et al., 2010; L. Wang et al., 2009).

The small molecule PRL inhibitors that have been identified to date have no selectivity for the different PRLs, which is likely due to the extremely high degree of sequence identity among them. Yet, resolved PRL-3 NMR structure and PRL-1/2 crystal structure suggest that there may be critical differences between these proteins that can be exploited in drug design, which will be discussed further below.

## 6. PRL structure informs drug discovery

### 6.1. PRL-3 structure

**Fig. 3.**
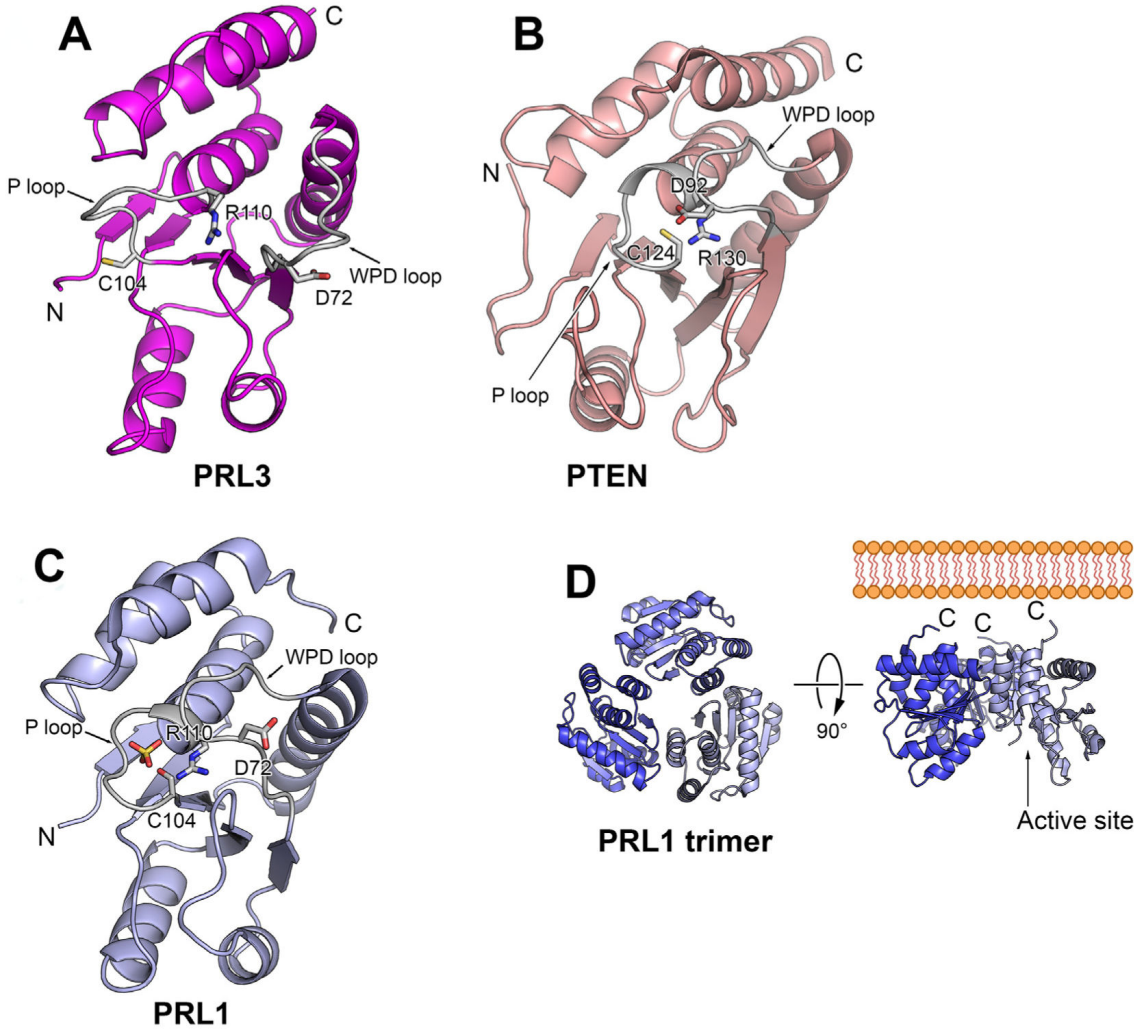
Comparison of PRLs structure and active site conformation. A) Cartoon view of PRL-1 monomer structure (Protein Data Bank code 1XM2) shows that PRL-1 adopts a closed conformation with the conservative aspartate residue (D72) in proximity to catalytic cysteine (C104). B) Cartoon view of PTEN reveals a close conformation in the active site (PDB ID 1D5R). The second domain of PTEN is omitted for clarity. C) Cartoon view of PRL-3 (Protein Data Bank code 1R6H) shows that PRL-3 adopts an open conformation with the conservative aspartate residue (D72) away from catalytic cysteine (C104). D) PRL exists as a trimer in the crystal (Protein Data Bank code 1XM2). Trimerization exposes C-terminal prenylation motif that anchors PRL-1 on the inner membrane. The active P-loop was located on the opposite side of the trimer.

PRL-3 structure was resolved independently using nuclear magnetic resonance (NMR) and shows consistent results (Kim etal., 2004; Kozlov et al., 2004). The apo structure, without ligand biding (PDB 1V3A), and a complex structure (PDB 2MBC) with the PTP inhibitor vanadate, of truncated PRL-3 (amino acid 1–162) was determined (Kim etal., 2004). The complex structure of a different truncated PRL-3 (amino acid1–169) with a generic phosphate ligand from PBS (PDB 1R6H) was also reported (Kozlov et al., 2004). In NMR analysis, residues His^103^ to Arg^110^, which are exactly the phosphatase signature motif HCXXGXXR, are not detectable due to the high conformational flexibility of the loop (Kim et al., 2004). The signals of those residues arose upon adding vanadate or phosphate to the PRL-3, suggesting that the ligand binding stabilized the flexible P-loop. Structure comparison between PRL-3, phosphatase of activated cells-1 (PAC-1) and phosphatase and tensin homolog (PTEN) showed that these phosphatases have similar overall folding. However, there are several critical conformational differences of the active sites between PRL-3 and these DUSPs. For example, PRL-3 adopts an open conformation with the general acid Asp ^72^ in a position away from catalytic Cys^104^, which is not the appropriate position for Asp^72^ to facilitate dephosphorylation (Fig. 3A). In contrast, PTEN shows a closed conformation with the general aid Asp being close to catalytic Cys (Fig. 3B), and PAC-1 adopts an intermediate conformation between open and closed. A study comparing free and vanadate-bound PRL-3 structures using NMR (K. W. Jeong et al., 2014) showed that PRL-3 was able to adopt a closed conformation upon vanadate addition, with Asp^72^, Cys^104^ and Arg^110^ close together. PRL-3 therefore likely undergoes substantial conformational rearrangement upon substrate binding, which pulls Asp^72^ near to catalytic Cys^104^.

Another structural analysis of PRL-3 (Kozlov et al., 2004) showed similar results in that PRL-3 contained the typical secondary structure of a DUSP, with close similarity to vaccinia H1-related (VHR), PTEN, and kinase-associated phosphatase (KAP). PRL-3 again showed an open conformation, which may also partially explain the low phosphatase activity toward synthetic substrate. In addition, the catalytic pocket of PRL-3 is the shallowest among the all known phosphatases, suggesting a broad range of potential PRL-3 substrates (Kozlov et al., 2004). Both studies revealed that an intra-molecular disulfide bond formed between catalytic Cys^104^ with nearby Cys^49^, which is conserved in all PRLs. The formation and regulatory role of this disulfide bond in PRL activity was validated in the subsequent study of PRL-1, in which the disulfide linkage was detected by mass spectrometry and non-reducing SDS PAGE. More importantly, catalytic activity of PRL-1 was blocked by H_2_O_2_, the reactive oxygen species that breaks disulfide bonds (Sun et al., 2005).

Importantly, all PRL-3 proteins used in structural determinations were truncated and excluded the prenylation motif to increase solubility of PRL-3 protein for NMR analysis. It is not clear that how the PRL-3 prenylation motif affects its conformation.

### 6.2. Critical structural differences between PRLs

PRL-1 crystal structure was resolved independently by two different groups (D. G. Jeong et al., 2005; Sun et al., 2005) with uniform results, although the PRL-1 proteins used for structure analysis were slightly different. The Jeong laboratory utilized a truncated human PRL-1 containing residues 4 to 163 and a C104S mutation for better crystallization (PDB 1XM2). Zhong-yin Zhang's group used a rat PRL-1 containing residues 1–160 only (without prenylation motif) as well as the C104S mutant to obtain three structures: PDB 1×24, which shows PRL-1 as a monomer; PDB 1ZCK, the structure of selenomethionine (SeMet)-substituted PRL-1, in which incorporation of SeMet into the protein helps solve the phase problem in crystallography to facilitate structure elucidation; and PDB 1ZCL, the structure of PRL-1 C104S mutant in complex with sulfate. Both PDB 1ZCK and PDB 1ZCL revealed PRL-1 as trimers (Fig. 3C-D).

The apo structure of PRL-2 has not been resolved yet. However, the crystal structure of the complex of PRL-2 and the cystathionine-beta-synthase (CBS)-pair domain of the Mg^2+^ transporter CNNM3 has been reported. The superimposition PRL-2 structure in this complex (PDB code: 5K22) with the crystal structure of PRL-1 (PDB code: 1XM2) suggests that there are no major conformational differences between these proteins (Gulerez et al., 2016). Further evidence is needed to determine if PRL-2 is also a trimer.

The PRL-1 crystal structure shows some similar features to that of PRL-3. For example, the topology of PRL-1 is similar to the catalytic domain of several DuSPs, including cell division cycle-14 (CDC14), KAP, MAPK phosphatases (MKP) and PTEN. There is also a disulfide bond between catalytic Cys^104^ and Cys^49^. Like PRL-3, the catalytic active site of PRL-1 is more shallow and open compared with other classes of PTPs.

However, surprisingly, there are some significant differences between the PRL-1 crystal structures and PRL-3 NMR structures, despite of the high degree of amino sequence identity between the proteins. First, they adopt different conformations of P-loop and WPFDD loop. PRL-1 crystal structure shows a close conformation of active site with the general acid Asp^72^ in proximity to catalytic Cys^104^, which is the optimal conformation for catalysis (Fig. 3C). However, as discussed above, the PRL-3 structure shows an open conformation with Asp^72^ being away from Cys^104^ (D. G. Jeong et al., 2005).

Secondly, the oligomeric state of PRL-1 and PRL-3 may be different. PRL-3 appears as a monomer in solution while PRL-1 exists as a trimer in 4 of the 5 crystals reported, which is unusual in other PTPs. The C-terminal of each PRL-1 monomer is positioned on the same surface of the trimer while the active sites are located to the other opposite site of the trimer, pointing to the outside of the trimer (Fig. 3D). However, direct evidence to support that PRL-1 exists as a trimer in solution or inside the cells is still limited. The crosslinking of over-expressed full length PRL-1 shows the existence of trimer (D. G. Jeong et al., 2005), but crosslinking of over-expressed proteins may generate false positive results due to random interaction of concentrated proteins. Dynamic light-scattering experiments showed that the C-terminal-truncated human PRL-1 protein exists as monomers at 1 mg/ml concentration and mixture of dimers and trimers at 7 mg/ml concentration (D. G. Jeong et al., 2005). The C-terminal-truncated rat PRL-1 shows as monomers at concentrations of 0.1–3 mg/ml (Sun et al., 2005). Interestingly, despite the high degree of sequence identity to PRL-1, PRL-3 exists only as a monomer in NMR experiments, even at the extremely high protein concentrations of 19.5 mg/ml and 58.5 mg/ml, respectively (Kim et al., 2004; Kozlov et al., 2004). Both PRL-3 and PRL-1 purified proteins were truncated in these analyses, and it remains unclear whether the C-terminus affects the oligomeric states of PRLs or not. Currently, there are no reports of the trimer formation of endogenous human PRLs.

Interestingly, there is some biological evidence implicating that the functional unit of PRL-1 is a trimer. Disruption of trimer formation of PRL-1 by mutation of key residues involved in the trimer interface (Gly 97 or Thr13) did not affect PRL-1 catalytic activity and subcellular localization, yet overexpression of trimerization-defective PRL-1 mutants (G97R or T13F) in human embryonic kidney (HEK) cells abolished the phenotypic consequences seen with overexpression of the wild type PRL-1, such as enhanced cell growth and migration (Sun et al., 2007). Additionally, a small molecule PRL inhibitor that was developed based on ability to disrupt the PRL-1 trimer showed anti-cancer properties both *in vitro* and *in vivo* (Bai et al., 2016). The crystal structure of PRL-1 in complex with a PRL trimer disruptor, named analog 3 of compound 43, was recently released in the Protein Data Bank (PDB 5BX1), which shows that analog 3 binds to the backside of PRL-1, mostly on one interaction face of trimer (Bai et al., 2016).

Whether PRL-1 and PRL-3 have different oligomeric states needs to be further validated. The open conformation of PRL-3 implicates that substantial conformational re-arrangement is necessary upon substrate binding. Perhaps the conformational state of PRL-1 is stabilized by trimerization, and if so, the different conformational re-arrangements between PRL-1 and PRL-3 may provide important information that will be useful in the design of PRL inhibitors with selectivity between them.

### 6.3. Structure of PRL-CNNM complex

CNNMs are magnesium transporters containing four homologs (CNNM1-4), which were identified as interacting partners of PRLs very recently (S. Hardy et al., 2015; Kostantin et al., 2016; H. Zhang et al., 2017). In these studies, PRLs were shown to act as pseudo-phosphatases and bind to CNNMs to increase intracellular magnesium concentration. High magnesium concentration is reported to relate to tumorigenesis and tumor progression based the studies on cultured cells, animal models and human samples (Castiglioni & Maier, 2011). Recent work has shown that PRLs may regulate intracellular magnesium *via* CNNM. One study suggests that the interaction between PRL and CNNM increases magnesium influx (S. Hardy et al., 2015), while a second proposes that the interaction blocks magnesium efflux (Funato et al., 2014). The exact mechanism through which PRLs may regulate magnesium concentration in the cells is still unknown and needs further investigation.

**Fig. 4.**
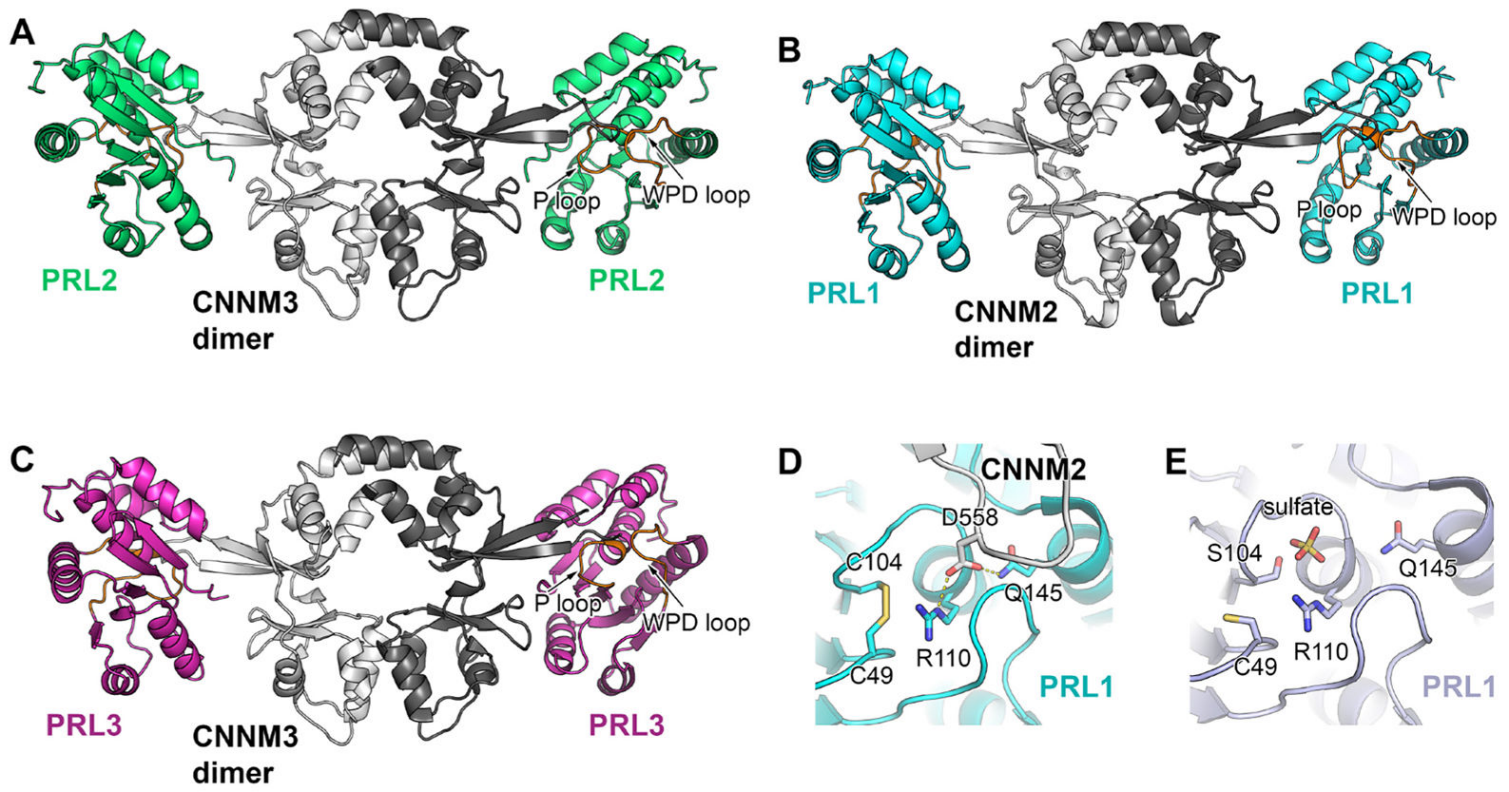
Structure of PRL-CNNM complex. A) Crystal structure of PRL-2 in complex with the CNNM3 CBS-pair domain (Protein Data Bank code 5K22) reveals a tetramer, where CNNM3 forms a dimer and binds to a PRL-2 protein at each side of the dimer. The interaction happens between the extended loop of CNNM3 and active site of PRL-2.B) Crystal structure of PRL-1-CNNM2 (Protein Data Bank code 5MMZ) and C) PRL-3-CNNM3 complex (Protein Data Bank code 5TSR) show similar interaction. D) and E) Comparison of PRL-1 active site conformation in the complex with CNNM2 with the conformation binding to a sulfate group that resemble its phosphate reveals similar conformation re-arrangement.

Crystal structures of PRL-CNNM complexes were resolved by several different labs, including PRL-1-CNNM2 (Gimenez-Mascarell et al., 2017), PRL-2-CNNM3 (Gulerez et al., 2016; H. Zhang et al., 2017) and PRL-3-CNNM3 (H. Zhang et al., 2017). The first resolved complex structure is formed between human PRL-2 and the cystathionine-(β-synthase (CBS) pair domain of CNNM3 (PDB 5k22) (Gulerezet al., 2016). CNNM3 and PRL-2 form a tetramer, where CNNM3 is a dimer and binds to two independent PRL-2 molecules at each side of the dimer (Fig. 4A). The interaction between CNNM3 and PRL-2 occurs between the extended loop of CNNM3 and active site of PRL-2. Specifically, Asp^426^ of CNNM3, which is functionally validated to be essential for high affinity binding to PRL-2, fits into the catalytic pocket formed by the P-loop and WPFDD loop of PRL-2. The fact that presence of wild-type CNNM3 inhibited PRL-2 phosphatase activity in an *in vitro* phosphatase assay while binding-deficient CNNM3 had no effect on PRL-2 activity suggests that CNNM3 might be a pseudo-substrate of PRL-2 (Gulerez et al., 2016). In addition, phosphorylation of the PRL-2 catalytic cysteine^104^-blocked CNNM3 binding to PRL-2 (H. Zhang et al., 2017), suggesting that the catalytic cysteine is necessary for their interaction. The pseudo-phosphatase hypothesis of PRLs is supported by the observation that binding of CNNM2 to PRL-1 decreases its phosphatase activity *in vitro* (Gimenez-Mascarell et al., 2017).

The crystal structure of PRL-1 and CNNM2 CBS pair-domain complex (PDB 5LXQ and PDB 5MMZ) (Gimenez-Mascarell et al., 2017) showed a very similar structure to that of the PRL-2 and CNNM3 complex discussed above (Fig. 4B). The PRL-1 and CNNM2 complex is also present as a hetero-tetramer and Asp^558^ in CNNM2, the homolog of Asp^426^ in CNNM3, and sits in the catalytic cavity of PRL-1 upon binding. Mutagenesis of Asp^558^ also confirmed that this Asp residue is required for the binding between PRL-1 and CNNM2. Surprisingly, the structural data resolved in PRL-CNNM complex suggests a PRL-1 trimer is sterically prohibited in the PRL-1 and CNNM2 complex, as trimerization would cause clashes with the associated CNNM2 protein (Gimenez-Mascarell et al., 2017). This analysis suggests that PRL-1 is unlikely to exist as trimer in the PRL-1-CNNM complex.

One critical difference between the different PRL-CNNM structures is the different PRL conformation present upon CNNM binding. In the PRL-2 and CNNM3 complex, no major conformational change of PRL-2 was found upon CNNM binding by comparing to the PRL-1 crystal structure (PDB code: 1XM2) due to lack of apo PRL-2 crystal structure (Gulerez et al., 2016). However, PRL-1 showed conformational re-arrangement upon CNNM2 binding (Gimenez-Mascarell et al., 2017). Specifically, the P-loop and WPFDD loop of PRL-1 collapsed toward the catalytic pocket to accommodate the interacting residues of CNNM2. The resulting closed conformation blocked the access to PRL-1 substrates, which might explain that CNNM2 association of PRL-1 decreased phosphatase activity *in vitro*.

Interestingly, although the biological function of PRL-1, in association with CNNM2, is catalysis-independent, the conformational re-arrangement of PRL-1 upon CNNM2 binding is actually similar to that of substrate binding (Fig. 4D-E). Therefore, the different roles of PRLs, as either a phosphatase or as an inhibitory binding partner of CNNM, seem to be exclusive. How these different roles of PRLs are regulated in the cells remains to be explored.

The recent report describing new crystal structures of PRL-3 in association with CNNM3 (PDB code: 5TSR) further supports the notion of PRLs acting as pseudo-phophatases to regulate the magnesium transport capability of CNNM (H. Zhang et al., 2017). The critical amino acid residues for binding CNNM in PRLs have been determined by mutagenesis, including catalytic residues Cys^104^ and Arg^110^ and non-catalytic residues Leu^108^ and Arg^138^. These CNNM binding-defective PRL mutants also showed decreased phosphatase activity *in vitro* with the exception of the PRL-3 R138E mutant, which showed unaffected phosphatase activity but weaker binding affinity for CNNM3.

In total, the PRL-CNNM complex structures provide molecular evidence for an entirely novel mechanism for the role of PRLs in tumor progression, namely by regulating intracellular magnesium.

### 6.4. Implications of PRL structure in inhibitor identification

PRL structures suggest they may be more amenable to small molecule inhibition than other classes of PTPs. For example, one of the challenges in developing PTP inhibitors is that the excessive positively charged active P-loop of PTPs can generally only accommodate strongly negatively charged inhibitors, which have poor cell permeability and low bioavailability (He, Yu, Zhang, & Zhang, 2014; Stanford & Bottini, 2017). However, the P-loops in PRLs are remarkably hydrophobic, suggesting that they may have preference for more hydrophobic substrates or inhibitors, which would have better cell membrane permeability.

Another obstacle for targeting PTPs is that the active site is highly conserved, making the development of selective orthosteric inhibitors very difficult. Several unique structural features of PRLs show promise to overcome this problem. For example, PRLs have a more shallow and open catalytic pocket than other PTPs, implicating different substrate specificity. The regulatory role of the disulfide bond between the catalytic Cys^104^ and Cys^49^ in PRLs activity also suggests that molecules regulating the formation of disulfide bond may specifically affect PRL activity. The unique CAAX prenylation motif and possible intrinsic trimerization of PRLs may also provide different targets for the development of inhibitors specific to PRLs over other PTPs. As discussed in Table 1, a PRL-1 trimer disruptor, compound-43, was successfully developed and showed an inhibitory effect on PRL-1 oncogenic activity *in vitro* and in a murine xenograft model of melanoma. However, whether this compound also inhibits PRL-2/3 has not been reported yet.

The open and inactive conformation of PRL-3 is significantly different from that of PRL-1, which is in a closed/active conformation. It is very likely that PRL-3 will go through substantial conformation rearrangement upon substrate binding while PRL-1 may not (D. G. Jeong et al., 2005; Kim et al., 2004; Kozlov et al., 2004; Sun et al., 2005). The apo structure of PRL-2 has not yet been defined and its conformation in the complex with CNNM3 also shows a closed/active conformation similar to PRL-1. Therefore, the possible conformational rearrangement and stabilization upon substrate binding observed in PRL-3 suggests that allosteric inhibitors that can bind to the inactive conformation of the PRLs to prevent conformational rearrangement may be effective PRL inhibitors.

Finally, the novel role of PRLs in binding CNNM to regulate magnesium level provides a new starting point to develop inhibitors that can disrupt this association.

## 7. Other strategies for targeting PRLs

Regulations of PRL expression and activities occurs at many levels, including gene amplification, as well as at the transcriptional, translational and post-translational levels, and has been reviewed (Rubio & Kohn, 2016). These types of regulatory mechanisms are now being considered as targets for the development of novel PTP inhibitors (Yu & Zhang, 2018); it would be beneficial to apply similar strategies to future efforts in PRL inhibitor development.

For example, redox regulation of catalytic cysteine in PTPs has been recognized to play an important role in controlling PTP activity (Jeroen, Markus, Rinesh, & Arne, 2014). When the catalytic cysteine (Cys-SH) of PTP1B is converted into sulfenic acid (Cys-SOH) by intracellular reactive oxygen species, the PTP loses its phosphatase activity, as Cys-SOH is no longer a nucleophile. This oxidation process is reversible, although the sulfenic acid can be further oxidized to an irreversible form. Several small molecules have been developed to target the oxidized PTP1B intermediate, including sulfone-stabilized caranions, 1.3-diketone derivatives, and dimedone (Yu & Zhang, 2018). PRLs are subject to similar redox regulation, making this a viable strategy for inhibiting PRL function.

PTP activity is also regulated by allosteric activation. For example, extensive biological and structural experiments suggest that protein kinase phosphatase 3 (MKP3) goes through a substantial conformational arrangement to form a closed/activated catalytic loop upon binding to its physiological substrate ERK2 (Fjeld, Rice, Kim, Gee, & Denu, 2000; Yu & Zhang, 2018). A small molecule MKP3 inhibitor, (E)-2-benzylidene-3-(cyclohexylamino)-2,3-dihydro-1H-inden-1-one (BCI), was identified in a transgenic zebrafish drug screen (Molina et al., 2009), and further study revealed that BCI blocked the conformational rearrangement of MKP3 upon substrate binding, specifically preventing the D262 residue to be pulled close to the P-loop (Korotchenko et al., 2014). As discussed above, PRL-3 is very likely to go through substantial conformation rearrangement upon substrate binding while PRL-1/2 may not. Targeting the critical residues for conformational rearrangement in PRL-3 may lead to the identification of specific PRL-3 inhibitors.

Aside from targeting regulatory mechanisms, an alternate strategy to specifically target individual PRLs lie in PRL antibodies (mAb). Thus far, antibodies have shown good selectivity among different PRLs *in vivo*. For example, the formation of metastatic tumors that overexpress PRL-1 in mice was only blocked by PRL-1 mAb but not PRL-3 mAb. Consistently, only PRL-3 mAb but not PRL-2 mAb has the capability to block the formation of PRL-3 overexpressing metastatic gastric tumor (Ke Guo et al., 2008). However, antibody-based therapy is conventionally used to target antigens on the cellular surface or secreted proteins, and PRLs are not presented on the surface of PRL-positive cancer cells (Guo et al., 2012). There are currently different lines of evidence indicating how PRL mAbs might target intracellular PRLs. One is that PRL mAb can enter intact cells. Antibody uptake is a proven phenomena in both normal and cancerous cells (Ke Guo et al., 2008). Another potential mechanism is that PRL-3 protein could be secreted, and act as bait for PRL mAbs, which is supported by the enrichment of PRL-3 mAb in PRL-3^+^ tumor microenvironments but not in PRL-3^−^ tumor microenvironments in a murine gastric tumor model (Thura et al., 2016). Whether PRL protein secretion is a general property of different tumors still needs to be validated. The exact molecular mechanisms by which PRL Ab inhibits the tumor formation remains elusive, although NK and B cells seem to be important for the anti-tumor effect of PRL mAbs (Guo et al., 2012).

Additionally, nanobodies have recently emerged as a potential cancer therapeutic. Derived from camelid heavy-chain only antibodies, nanobodies are comprised of a single antigen-binding variable domain and have several advantages over conventional antibodies, including a small size (~15 kDa), stability under stringent conditions, are nonimmunogenic, and have high specificity and affinity. Nanobodies are able to penetrate the cell membrane (Van Audenhove & Gettemans, 2016), making them ideal for targeting intracellular proteins. Development of nanobodies that specifically target individual PRLs, and either block their catalytic domain or lock them in an open conformation, is a potential therapeutic strategy worth exploring.

## 8. Conclusions

The oncogenic role of PRLs is well established, and these phosphatases are now considered attractive therapeutic targets in cancer. Before we move from bench to bedside, there are still critical questions that need to be answered. For example, what are the physiological substrates of PRLs? What are the substrates in malignant conditions? How are PRLs involved in regulating tumor progression? A deeper understanding of PRL structure will allow us to define the ways PRL proteins interact with their substrates, and will lead to the development of specific small molecule inhibitors. In turn, these small molecules will facilitate more in-depth study of PRL function and ultimately lead to drug development.

## Figures and Tables

**Table 1:** PRL inhibitors.

Inhibitor	Potency and selectivity	Discovery	Cellular efficacy	Reference
Pentamidine	Similar activity against PRL-1/2/3: IC_50_ < 0.277 μg/ml using peptide substrate; also shows activity against other phosphatases.	The clue comes from that pentamidine has similar anti-leishmania action as SSG, which has anti-cancer activity *via* inhibiting PTPs.	Inhibits growth of five human cancer cell lines with PRL expression within the concentration ranges 0.3-5 μg/ml; inhibits growth of WM9 human melanoma tumors in nude mice and results in tumor necrosis.	(Pathak et al., 2002)
Thienopyridone	PRL-1: IC_50_ = 173 nM PRL-2: IC_50_ = 277 nM PRL-3: IC_50_ = 128 nM Shows selectivity over 11 other PTPs using DiFMUP substrate.	High-throughput screening of the Roche chemical library to search for molecules that inhibits PRL phosphatase activity using peptide substrate.	Induce p130Cas cleavage and apoptosis in Hela and RKO cells; inhibits anchorage-independent growth in RKO and HT-29 cells; inhibits HUVEC cells migration but not proliferation.	(Daouti et al., 2008)
Iminothienopyridinedione 13 (JMS-053)	PRL-1: IC_50_ = 50 nM PRL-2: IC_50_ = 53 nM PRL-3: IC_50_ = 18 nM Minimal effect on 25 other phosphatases at 1 μM using DiFMUP substrate.	Identified during the process of SAR study of thienopyridone to increase its stability and reduce its potential toxicity.	Inhibit migration, spheroid growth and RhoA activity in human ovarian cancer cells; reduce growth of drug-resistant ovarian cancer in a murine xenograft model.	(McQueeney et al., 2018; Salamoun et al., 2016)
Rhodanine and its derivatives	Inhibit human PRL-3 activity with IC_50_ range 0.9-9.5 μM; compound 5e is the most active one with IC_50_ 0.9 μM against PRL-3; not tested on other PRLs and PTPs (DiFMUP).	Rhodanine skeleton was identified by high throughput screening of chemical library of Korean Chemical Bank and 14 derivatives were synthesized for SAR study.	Compound 5e reduces invasiveness of B16F10 cells *in vitro*.	(Ahn et al., 2006)
CG-707 and BR-1	Inhibit human PRL-3 activity: CG-707: IC_50_ = 0.8 μM BR-1: IC_50_ = 1.1 μM Minimal effects on 9 other PTPs (DiFMUP). Suppress cancer cell migration: CG-707: IC_50_ = 5 μM BR-1: IC_50_ = 7 μM	Using cell-based assay to screen rhodanine derivatives.	Inhibit the migration and invasion of cancers that express PRL-3 without affecting proliferation; change the expression of EMT markers.	(Min et al., 2013)
Analog 3	Inhibit human PRL-3 activity PRL-3: IC_50_ = 31 μM. Shows acceptable selectivity against PTP1B, TCPTP and VHR and no selectivity against PRL-1 and PRL-2 (DiFMUP).	Ligand based virtual screening of Zinc database combined with SAR study and biochemical screening.	Specifically inhibits migration of cells that express PRLs in a dose-dependent manner and does not affects proliferation of HEK cells at 50 μM.	(Hoeger et al., 2014)
Compound-43 and several analogs.	Not phosphatase inhibitors, Trimerization disruptors 30 mg/kg cmpd-43 inhibits melanoma xenograft tumor growth; ~2 μM cmpd-43 suppress 50% MeWo cells survival	Sequential structure-based virtual screening of compounds of Asinex and ChemBridge subsets in the ZINC database.	Specifically inhibit cell proliferation and migration of PRL-1 overexpressing cells; suppress MeWo cells proliferation and migration, inhibit melanoma xenograft tumor growth.	(Bai et al., 2016)
Curcumin	Inhibit human PRL-3 activity PRL-3: IC_50_ = 31 μM Shows acceptable selectivity against PTP1B, TCPTP and VHR and no selectivity against PRL-1 and PRL-2	Extracted from spice turmeric, has been known to be able to induce apoptosis of cancer cells and suppress cell migration and angiogenesis.	Inhibits mRNA expression of PRL-3 and partial PRL-2; specifically inhibits adhesion and migration of cancer cells with high PRL-3 expression; inhibits growth and metastasis of xenograft tumors in mice.	(L. Wang et al., 2009)
Ginkgetin and sciadopitysin	Inhibited PRL-3 activity: gfinkgetin: IC_50_ = 50 μM sciadopitysin: IC_50_ = 25.8 μM Not tested on other PTPs and PRLs	Bioflavonoids identified in the MeOH extract of the young branches of *Taxus cuspidata*	Reduce invasiveness of B16F10 cells *in vitro*	(Choi et al., 2006)
Natural anthraquinone compounds: compound 1 and 2	Inhibited PRL-3 activity: Compound 1: IC_50_ = 5.2 μg/ml Compound 2: IC_50_ = 1.3 μg/ml. Not tested on other PRLs and PTPs.	Extracted from the roots of Rubia akane.	Compound 2 was shown to inhibit migration of DLD-1 cells but not proliferation.	(Moon et al., 2010)
PRL-1 and PRL-3 mAb (mouse); Chimeric mouse and human PRL-3 mAb; PRL-3-zumab	High selectivity: PRL-1 and PRL-3 mAb only prevents metastatic tumor formation of cells that express respective PRL. PRL-3-zumab is more potent than 5-FU, a first-line chemotherapeutic drug for gastric cancer.	Generated using hybridoma technology and their specificity was confirmed.	Efficiently and specifically block metastatic tumors formation of cells overexpressing PRL; also inhibit tumor formation of cancer cells expressing endogenous PRL; prevents recurrence of PRL-3 positive tumors after surgery.	(Guo et al., 2012; Ke Guo et al., 2008; Thura et al., 2016

## Abbreviations:

CBS: cystathionine-beta-synthase
CHO: Chinese hamster ovary
CNNM: magnesium transporters of cyclin M
EMT: epithelial–mesenchymal transition
HMVEC: human microvascular endothelial cells
mAb: monoclonal antibody
NMR: nuclear magnetic resonance
PDB: protein data bank
PRL: phosphatase of regenerating liver
PTK: protein tyrosine kinase
PTP: protein tyrosine phosphatase
PTEN: phosphatase and tensin homolog
SAR: structure–activity relationship
SHP2: protein tyrosine phosphatase N11
SSG: sodium stibogluconate
TCPTP: T-cell protein tyrosine phosphatase
VHR: vaccinia H1-related phosphatase
